# Acceptability of HIV self-testing: a systematic literature review

**DOI:** 10.1186/1471-2458-13-735

**Published:** 2013-08-08

**Authors:** Janne Krause, Friederike Subklew-Sehume, Chris Kenyon, Robert Colebunders

**Affiliations:** 1 Institute of Tropical Medicine and International Health, Charité-Universitätsmedizin, Berlin, Germany; 2 loveLife, HIV prevention and youth development NGO, Johannesburg, South Africa; 3 Institute of Tropical Medicine, Antwerp, Belgium; 4 University of Cape Town, Cape Town, South Africa; 5 University of Antwerp, Antwerp, Belgium

**Keywords:** HIV, Testing, Alternative, Counselling, Self-test, Home test

## Abstract

**Background:**

The uptake of HIV testing and counselling services remains low in risk groups around the world. Fear of stigmatisation, discrimination and breach of confidentiality results in low service usage among risk groups. HIV self-testing (HST) is a confidential HIV testing option that enables people to find out their status in the privacy of their homes. We evaluated the acceptability of HST and the benefits and challenges linked to the introduction of HST.

**Methods:**

A literature review was conducted on the acceptability of HST in projects in which HST was offered to study participants. Besides acceptability rates of HST, accuracy rates of self-testing, referral rates of HIV-positive individuals into medical care, disclosure rates and rates of first-time testers were assessed. In addition, the utilisation rate of a telephone hotline for counselling issues and clients` attitudes towards HST were extracted.

**Results:**

Eleven studies met the inclusion criteria (HST had been offered effectively to study participants and had been administered by participants themselves) and demonstrated universally high acceptability of HST among study populations. Studies included populations from resource poor settings (Kenya and Malawi) and from high-income countries (USA, Spain and Singapore). The majority of study participants were able to perform HST accurately with no or little support from trained staff. Participants appreciated the confidentiality and privacy but felt that the provision of adequate counselling services was inadequate.

**Conclusions:**

The review demonstrates that HST is an acceptable testing alternative for risk groups and can be performed accurately by the majority of self-testers. Clients especially value the privacy and confidentiality of HST. Linkage to counselling as well as to treatment and care services remain major challenges.

## Background

Globally, only about one third of young people know their HIV status, which is far below the United Nations General Assembly Special Session target of 95% [1]. Increasing access to and uptake of HIV testing is critical to reduce the incidence of HIV and to improve access to treatment and support for seropositive people. People who are aware of being HIV-positive are less likely to engage in sexual risk behaviour [2] and people who receive antiretroviral treatment (ART) and adhere to it are less likely to be infective to others [3]. Both will decrease transmission of the virus and impact on the epidemic. Moreover, stigma is likely to decline the more people know their serostatus as this could enhance a ´normalisation` of the diagnosis [4,5]. In order to increase global testing rates and to ensure early access to treatment, a further exploration of new HIV testing options should be a research priority. HIV self-testing (HST), which refers to the performance of a simple saliva or blood-based self-test similar to a pregnancy test in the privacy of clients` homes or any other place that suits clients, could have the potential to bypass some barriers currently deterring people from testing. HST can either be performed using home sample collection kits or home self-testing. With real do-it-yourself tests, the client collects a sample, usually saliva or a blood spot from a finger prick, runs the rapid test and reads the test results. Pre-test information is provided in written form or online on websites of manufacturers and post-test counselling is provided via telephone hotlines. With home sample collection kits, the client takes a sample, saliva or a dried blood spot (DBS), which is then mailed to a laboratory. Afterwards, the client receives the result by phone or mail, which is linked to post-test counselling and, in case of a reactive test, is referred to follow-up services.

Community outreach HIV counselling and testing (HCT) programs like mobile clinics and door-to-door testing are among the tested alternative strategies and have already significantly improved testing uptake and reached higher rates of first-time testers than facility-based testing services in Sub-Saharan Africa [6-11]. International experts believe that HST could be the next step [12,13]. Highly accurate self-test kits exist. The OraQuick® In-home HIV test (OraSure Technologies, Inc.) has a sensitivity of 92% and a specificity of 99.9% and was recently approved by the U.S. Food and Drug Administration for over-the-counter sale [14]. It is now available at major American pharmacies [15]. A commonly raised concern as regards HST is that testing without counselling may result in adverse psychological outcomes [16]. In addition, linkage to care constitutes a particular challenge [17]. Further research is needed to answer critical questions on acceptability, feasibility, safety and cost-effectiveness of HST. As a first step, we performed a systematic literature review with the main objective to assess the acceptability of HST by key populations. Secondary objectives were to review the accuracy of HST, linkage to care of clients with a reactive HIV self-test, disclosure rates of self-testers, utilisation rates of telephone hotlines for counselling and qualitative attributes of HST.

## Methods

### Study design

We performed a systematic literature review using the guidelines for Preferred Reporting Items for Systematic Reviews and Meta-Analyses [18]. We included all studies in which HST was offered to and performed by study participants. Utilised HIV test devices included home sample collection tests with performance of standard Western blot and real self-tests based on blood or saliva rapid tests. We included the two HST methods, as our main interest was acceptability of HIV testing in privacy, which is central to both testing strategies. In the absence of international recommendations about HST, no standardised procedure for HST was required.

### Search strategy and restrictions

**Figure 1:**
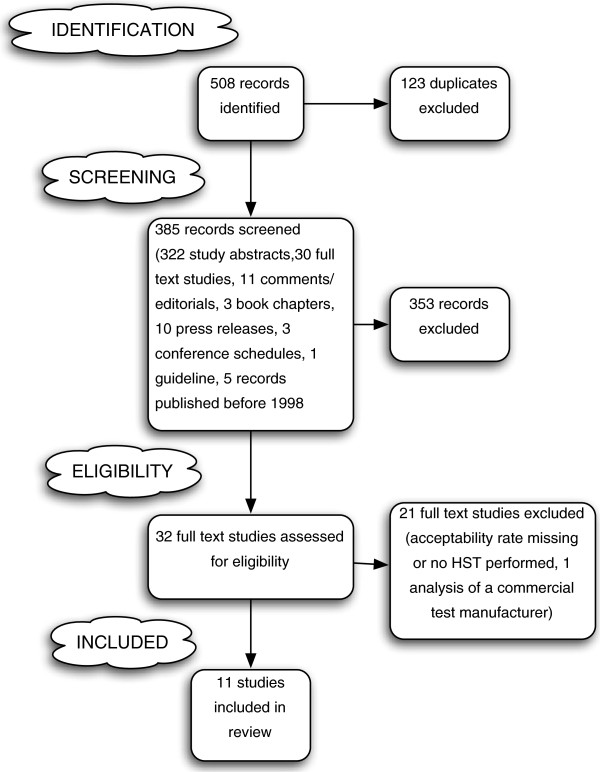
**Selection process of HIV self-testing studies following the PRISMA methodology**[18]**.**

Pubmed, Embase, ScienceDirect, The Cochrane Library and the Global Health Database were systematically searched for matching manuscripts and a comprehensive Google search was performed for grey literature. Search terms included ´HIV` AND ´self-test` OR ´self-test` OR ´self-testing` or ´home test` OR ´home sample collection test`. Preferably peer-reviewed studies published in English between 1998 and October 2012 were included. Conference abstracts were eligible but no comments, editorials and unpublished reports. Because of the limited number of published studies, the search was not restricted to a specific study type. The different phases of the study selection process are presented in a flow diagram (Figure 1).

### Data analysis

The study focussed on the outcome ´acceptability of HST`. The acceptability rate was defined as the proportion of all people approached to participate in a study, who eventually performed HST. Insights around the following topics were also extracted whenever possible: accuracy of HST, here defined as the proportion of self-test results in agreement with confirmatory test results performed and interpreted by trained health staff (invalid test results have been included as discordant test results), referral rate of those who tested seropositive into medical care, disclosure rate of self-test results, rate of first-time self-testers and utilisation rate of a telephone counselling hotline. Besides extraction of these rates, each study was reviewed for various qualitative aspects of HST. Because of the heterogeneity of included studies regarding study design and study populations, no meta-analysis has been performed. Study characteristics as well as outcomes are presented in tabular form (Tables 1 and 2, Table 3 respectively).

**Table 1:** Studies evaluating HIV self-testing (HST) in high-income countries

**Study**	**Carballo-Diéguez et al.,**[19]	**Carballo-Diéguez et al.,**[20]	**De la Fuente et al.,**[21]	**Belza et al.,**[22]	**Katz et al.,**[23]	**Gaydos et al.,**[25]	**Lee et al.,**[27]	**Spielberg et al.,**[28]	**Osmond et al.,**[29]
**Objective**	Acceptability of oral fluid HST and willingness to use prior to sexual intercourse among high-risk MSM	Acceptability of oral fluid HST prior to sexual intercourse among sexual partners of MSM	Feasibility of blood-based HST and interpretation of results by laypersons	Feasibility of blood-based HST by laypersons	Acceptability and ease of use of oral fluid HST compared to clinic-based HCT among MSM	Feasibility and accuracy of oral fluid and blood-based HST by laypersons compared to trained staff	Acceptability and feasibility of blood-based HST among high-risk populations	Feasibility and acceptability of bimonthly oral fluid and DBS HSC among high-risk populations	Acceptability and feasibility of oral fluid HSC for HIV testing among MSM
**Location**	New York City, USA	New York City, USA	Gay districts and university sites, Madrid, Spain	see De la Fuente et al., [21]	USA	Baltimore, USA	Singapore	USA	San Francisco, Los Angeles, New York City, Chicago, USA
**Population**	57 high-risk MSM	140 sexual partners of high-risk MSM	313 HCT clients of mobile testing units	267 HCT clients of mobile testing units	68 HIV-negative high-risk MSM	565 patients of emergency departments	420 HIV-positive- and at-risk clients of communicable disease and STI centres	297 participants of HIV Network for Prevention Trials	615 participants of Urban men´s health study
*Sex*	100% male	100% male	69% female, 29% male	41% female, 51% male	100% male	59% female, 41% male	9% female, 75% male	20% female, 80% male	100% male
*Median age*	34 years	n.s.	n.s.	n.s.	39 years	38,4 years	33 years	36 years	n.s.
*Ethnicity*	39% Latino, 32% Black, 21% White, 7% Asian	n.s.	83% White, 16% Latino	83% White, 16% Latino, 1% n.s.	73% White, 13% Latino, 9% Black, 4% Asian	69% Black, 29% White	98% Asian	64% White, 18% Black, 12% Latino, 2% Asian	69% White, 12% Hispanic, 8% Black
*MSM*	100%	100%	35%	38%	100%	n.s.	9%	58%	100%
*IDU*	-	-	n.s.	n.s.	-	> 15%	n.s.	30%	-
*WAHR*	-	-	n.s.	n.s.	-	n.s.	n.s.	12%	-
**Acceptability of HST**	73,7%	81,5%	83%	77.9%	63.2%	84.6%	83.3%	81.1%	67%
**Incentives**	No	Yes	No	No	No	Yes	No	Yes	Yes

*n.s.* not specified, *MSM* Men who have sex with men, *IDU* Injecting drug users, *WAHR* Women at heterosexual risk.

**Table 2:** Studies evaluating HIV self-testing (HST) in low-income countries

**Study**	**Choko et al.,**[24]	**Kalibala et al.,**[26]
**Objective**	Use and accuracy of oral fluid HST in the general population	Acceptability and feasibility of free oral fluid HST among HCW
**Location**	High residential suburbs of Blantyre, Malawi	District and provincial hospitals in Kenya with variable degrees of pre-existing HIV related services
**Population**	298 of adult population and community peer group members	1081 HCW
*Sex*	52% female, 48% male	33% female, 67% male
*Median age*	26.5 years	n.s.
*Ethnicity*	n.s.	n.s.
*MSM*	n.s.	n.s.
*IDU*	n.s.	n.s.
*WAHR*	n.s.	n.s.
**Acceptability of HST**	87.2%	21.9%
**Incentives**	No	No

*n.s.* not specified, *MSM* Men who have sex with men, *IDU* Injecting drug users, *WAHR* Women at heterosexual risk.

**Table 3:** Outcomes of HIV self-testing (HST) studies

**Study**	**Carballo-Diéguez et al.,**[19]		**Carballo-Diéguez et al.,**[20]		**De la Fuente et al.,**[21]		**Belza et al.,**[22]		**Katz et al.,**[23]		**Choko et al.,**[24]		**Gaydos et al.,**[25]		**Kalibala et al.,**[26]		**Lee et al.,**[27]		**Spielberg et al.,**[28]		**Osmond et al.,**[29]	
**Sample size**	**57**		**140**		**377**		**267**		**68**		**298**		**565**		**1081**		**420**		**297**		**615**	
**Outcome**	N	%	N	%	N	%	N	%	N	%	N	%	N	%	N	%	N	%	N	%	N	%
**Acceptability rate**	42	73,7	101	72,1	313	83	208	77,9	43	63,2	260	87,2	478	84,6	237	21,9	350	83,3	241	81,1	412	67
**Accuracy rate**	-		-		288*	92	206**	99	-		256	98,4	476	99,6	-		158***	45,6	658	99	402	97,1
**Proportion of 1st-testers**	-		-		142	45,4	111	56,9	-		94	36,2	-		-		-		-		71	17,2
**Disclosure rate**	--		-		-		-		-		-		-		192	81	-		-			
**Telephone hotline utilisation rate**	-		-		-		-		-		-		-		1	0,4	-		228	94,6	172	41,7

*25 invalid test results.

**2 invalid test results.

***192 invalid test results.

## Results

Eleven studies met our inclusion criteria [19-29] Tables 1 and 2. The home sample collection method was performed in two studies [28,29], the remaining studies used either blood-based [21,22,25,27] or saliva-based [19,20,23] rapid tests. A very recent study from Singapore [30] on accuracy and acceptability of HST was not eligible for the review, as the study does not mention the proportion of all people approached who accepted HST and thus does not provide an acceptability rate. Yet, the study incorporated a survey on user acceptability after performance of HST to which we refer in the discussion section. Two studies were performed in Sub-Saharan Africa [24,26], six in the USA [19,20,23,25,28,29], two in Spain [21,22], and one in Singapore [27]. Study populations consisted of risk groups like health care workers (HCW) [26], men who have sex with men (MSM), injecting drug users (IDU), clients of sexually transmitted infection (STI) clinics, women with multiple sexual partners or HIV-seropositive sexual partners [19,20,23,27-29] and the general population [21,22,24,25]. Most included studies were observational and lacked a comparison group. Typically, a cross-sectional survey was performed. Only two studies consisted of a randomized controlled trial [23,28]. Outcomes are summarised in Table 3.

### Acceptability of HST

Acceptability of HST was high in most studies. Seventy per cent of all clients who were included in the reviewed studies performed HST. The acceptability rate ranged from 22% to 87% and was highest in Malawi where HST was offered at home with minimal supervision [24]. In the Malawian study, HST uptake was similar for both genders despite a significantly lower testing history among men. In Baltimore, overall acceptability of HST was high among hospital emergency department clients, but far more people (91% vs. 9%) opted for saliva-based HST compared to blood-based HST [25]. Spielberg et al. [28] demonstrated high initial interest to self-test (81%) but greater adherence in the oral fluid bi-monthly specimen collection arm compared to the DBS arm. In Spain, 78% [22] and 83% [21] of clients of mobile testing units agreed to HST. HST in these mobile testing units was especially attractive to gay people, young people, singles and people who had never tested before for HIV. At least two-thirds of an American high-risk MSM population reported use of HST with an average use of 1.6 times/per person [23]. 73% of MSM with frequent unprotected anal intercourse and changing sexual partners agreed to HST [19], as did 82% of their sexual partners [20]. As early as 2000 [29], acceptability of HST among MSM participating in the Urban Men´s Health study had been high (67%). Uptake among Kenyan HCW was substantially lower [26]. Only slightly more than a fifth (22%) of all approached HCW eventually performed HST, and only about one third (31%) of those HCW who participated in the post-intervention survey had performed HST. Among HCW who attended pre-test information sessions, acceptability of HST was as high as in other studies (75%). In a separate intervention session arm, in which the intervention team had actively formed groups to attend pre-test information sessions, 97% of HCW took a test kit, but there was no further investigation around how many of them subsequently performed the test. HCW were additionally offered to take test kits for their partners, which resulted in 55% of partners performing HST.

### Accuracy of HST

Overall, agreement between test results performed by laypersons and health staff was high with 86% of self-tests and 98% of home samples collected adequately. Disagreement between health staff and study participants` results was mainly caused by invalid test results due to performance errors. Overall, no false positive test results occurred and the few false negative or inconclusive test results were mainly related to misinterpretation of very faint lines. In Spain, self-testers performed tests as accurately as health staff with 99% of tests being valid results [22]. However, the proportion of valid HST results decreased to 92%, when demonstration of the testing procedure through trained health staff was omitted [21]. Accurate performance was associated with the level of education. Misinterpretation of photo results was generally rare (4,9%) and associated with older age, Latin American origin and lower educational levels. Although self-administered tests were performed very accurately in Malawi, overall sensitivity (97.9%) remained slightly below sensitivity claimed by the manufacturers and was even lower (96.4%) among those who were previously not known to be HIV-positive [24]. Test results of participants in Baltimore concurred in 99,6% with the results of the tests performed by health staff [25]. The study from Singapore [27] was the only one with high rates (54,4%) of invalid test results largely because most users failed to correctly transfer a blood sample with a capillary tube. Known HIV-positive participants were more likely to perform and interpret HST accurately.

### Utilisation of a counselling telephone hotline

Few studies provided information on this topic, two from Sub-Saharan Africa [24,26] and three from the USA [23,28,29]. Utilisation of a counselling telephone hotline differed markedly between studies and countries and was substantially lower in Sub-Saharan Africa than in the USA. African participants clearly expressed a need for face to face counselling and comprehensive post-test counselling services [24,26]. American high-risk individuals on the other hand strongly preferred post-test counselling by phone (95%) and the majority suggested that, in the case of repeated testing, pre-test information would only need to be given every 6–12 months [28]. Despite these findings, only 40% of MSM included in the Urban Men´s Health Study called for their results and 7% of those who declined to participate in the study expressed concerns on being given the result by phone [29]. American high-risk MSM used the 24-hours contact line only to request new test devices and called the study office for counselling issues [23].

### Disclosure of test results

The reviewed studies give little information on disclosure of test results among self-testers. Most HCW (81%) and partners (85%) discussed their test result with another person, mainly with their sexual partner [26]. Other persons of trust were colleagues and friends, but only 10% discussed their result with another HCW. Couple testing showed increased disclosure rates. Women in Malawi felt HST could make involvement of their husbands in testing easier and remove some of the burden of testing from their shoulders [24]. Disclosure of test results did not seem to be an issue for American high-risk MSM, as the vast majority agreed to partner testing and accordingly disclosed their status [20].

### Proportion of first-time testers

In the four studies [21,22,24,29] that investigated the proportions of first-time testers, the proportions of people who had never tested before were equal among those who finally performed HST and those who were eligible to participate in studies. This suggests a high uptake of HST among first-time testers. In both Spanish studies half of participants were testing for the first time [21,22]. In Malawi, 50% of seropositive cases were newly detected through HST [24]. Among American high-risk MSM who tested seropositive, 60% had previously been unaware of their HIV-status [20].

### Attitudes and opinions on HST

Despite very different individual, socio-economic and cultural backgrounds, emerging qualitative assessments of HST were similar among study populations. HST was perceived as highly confidential and private [24,26,28] and participants believed HST could give people more control over their health [25]. High-risk MSM thought HST would increase testing frequency [23] and HCW regarded HST to be a testing strategy that they could easily access and that would facilitate their decision to get tested including testing for the purpose of post exposure prophylaxis [26]. People generally thought HST was easy to perform especially when a saliva-based test was used [21,23-29]. Indeed, Spanish participants rated collection of blood drops as the most difficult step of HST and those with invalid test results were more likely to believe that instructions were complicated [21]. Rapid oral fluid testing was generally preferred to blood-based testing [25-28]. Participants who self-tested were likely to use HST for their next HIV test and stated they would recommend HST to family and friends [19-21,24-28]. Trust in HST was high [25], but there was also some scepticism on accuracy [26,27,29] and the limitation of adequate post-test counselling services [24,26-29]. Further, acceptance of HST was influenced by its costs although most participants were willing to pay a small to medium amount [21,23,26-29]. Whereas Kenyan health care workers opted for a small fee ´comparable to a pregnancy test` [26], European and American clients would accept higher costs: 20 Euros [21] and 10 to 40 USD [23], respectively. Moreover, the way in which HST kits are distributed impacts on their acceptability. Participants from the USA and Singapore opted for public sale of HST kits [20,27], such as at pharmacies, whereas participants from Malawi [24] preferred home-based distribution of self-testing kits. Kenyan HCW believed self-test kits should be universally available at health facilities or even distributed on a routine base [26].

## Discussion

Our reviewed studies demonstrated consistently high acceptability of HST, particularly with saliva based rapid tests, in all populations where so far the testing was evaluated. Acceptability was also high in the two home sample collection studies (81% [28] and 67% [29]). HST encouraged testing equally among both genders [24]. Moreover, it encouraged repeated testing and first-time testing in hard-to-reach groups [20,22,23]. Even in African countries with existing successful testing programmes HST increased the reach of HIV testing services: 41% of participants in Malawi had never tested before and 78% had tested longer than a year ago [24]. Further, HST seems to be an acceptable screening tool prior to sexual intercourse for MSM with high-risk sexual behaviour [19,20] despite its limitations related to the window period. Uptake of HST among MSM and their partners was high, HST was predominantly well appraised and a significant number of unknown seropositive cases were detected [20]. As such HST could detect and prevent HIV transmission in this population at least partly. In a recent study from Singapore [30], overall, 87.4% of participants would purchase an over-the-counter rapid test kit, with the highest proportion being among private clinic at-risk participants (92.4%).

With regard to Sub-Saharan African countries, acceptability rates of HST seem to be comparable to those of home-based HCT or provider-initiated HCT approaches [31-33]. However, high testing uptake of home-based HCT is often impaired through low rates of clients who finally learn their results [34,35]. The majority of reviewed studies do not report how many clients who tested reactive on HST consulted for obtaining a confirmatory test, thus we do not know how many self-testers really learn their result. Only 42% of MSM, who used the home sample collection method in the Urban Men´s Health Study [29] called the help line for their testing result and participants of the HIV Early Detection Study [28] were more willing to test for HIV with rapid tests than with the home sample collection method in order to avoid an unpleasant waiting period.

The acceptability of HST was lowest for Kenyan HCW [26]. The reason for this may be related to the low attendance rates of pre-test information sessions where HCW could receive HST kits. Acceptability was comparably high to other studies, once HCW attended pre-test information sessions. In some studies, willingness to test for HIV was a precondition for inclusion in the study [21,22,25,28]. Therefore, acceptability rates did not truly reflect participants` decision to get tested, but rather whether someone wanted to experience HST. In other studies, participants received incentives, which might also have impacted on their decision to self-test [20,25,28,29]. It is possible, that the Kenyan study gives a more realistic picture of the degree to which HST, if available in a country, could add to HCT uptake through existing testing strategies. In this case, an increase of 20% of HCW knowing their HIV status through HST could be regarded as a considerable achievement. Part of this benefit would be related to the high partner-testing rate and disclosure rate, that the study of Kalibala et al. [26] found.

Critics often question the feasibility of HST among laypersons. However, most participants performed HST accurately with little or no help of trained personnel [21,22,24,25]. There were no false positive results, which is remarkable as false positive results have been reported consistently in the context of rapid HIV tests especially in low-prevalence settings, highlighting the need for follow-up of clients for confirmatory testing [36-40]. The exact accuracy of HST in real-life settings remains to be determined. In several studies, clients were trained by professional staff prior to HST [22,23,26,28], performed the self-test under supervision [20-22,24] or had just received an oral fluid rapid test performed by trained personnel [25]. In Spain [21], the proportion of valid test results dropped from 99% to 92%, when the demonstration of the test through trained staff was omitted. In Malawi [24], 10% of self-testers, especially illiterate, less educated participants and women, requested the help of supervisors. Hence, accuracy is likely to be reduced, when clients depend only on themselves. User-friendliness of self-test devices is crucial to accurate performance. Studies by De la Fuente et al. [21] and Ng et al. [30] have demonstrated that depositing blood drops directly onto the test reactive strip (bypassing the use of a capillary tube for blood collection) improved the validity of HST. The now FDA-approved OraQuick® ADVANCE Rapid HIV-1/2 test seems to fulfil pegged requirements and reaches high accuracy in the hand of laypersons [41].

Our results support the theory that HST holds the potential to increase testing uptake. Nevertheless, some issues remain unanswered. Future research will also need to concentrate on other target groups that so far have been neglected in the context of HST like youth, women and couples. Furthermore, the organisation of counselling services and linkage to treatment and care has to date not been sufficiently investigated. Research from The Netherlands suggests that self-testers feel even more responsible for their health and show higher levels of self-efficacy than people who do not use self-tests [16]. Most studies included confirmatory testing of HST [20-22,24,25,27,28], none of the reviewed studies have, however, examined whether self-testers reliably seek medical care in case of a positive result and what effect HST could have on HIV transmission. In any case, it will be essential to link HST to adequate counselling and support services. However, various degrees of counselling and various counselling models are conceivable. Today it is considered that pre-test counselling can be minimal, merely providing the client with sufficient knowledge to give informed consent [42]. In fact, American high-risk individuals preferred optional counselling, telephone hotlines or written pre-test information to mandatory in-person counselling [43-45]. Further, in-person counselling is no guarantee for good quality: an evaluation of calls at the South African National AIDS helpline indicated that a substantial proportion of clients who received in-person counselling at VCT services did not understand the meaning of their result, or only received a written test result without any counselling [46]. HST is so far only linked to counselling telephone hotlines, but, especially in developing countries, telephone hotlines do often not seem to cover clients` needs when it comes to notification of results and adequate post-test counselling. In rural areas with limited telephone, group information sessions and close links to on-site social organisations could be a possible counselling and support strategy for self-testers [12,13]. A recent study from Malawi demonstrated that linking HST to home assessment and the initiation of HIV care can significantly increase both the disclosure of HIV status and the initiation of ART at a population level [47]. Two of the self-testing studies took place in mobile units in Spain [21,22]. Staff offered HST to clients asking for VCT and reached a significant number of people. Clients received counselling and performed HST under supervision of trained personnel at the mobile unit.

At the moment, HST is still of disputed legality in many countries, such as South Africa, and happening only informally [48]. Although international policies stress a public health approach to HTC and a move away from obligatory in-depth pre-test counselling, the World Health Organization has not as yet given any specific advice for the implementation of HST services [49]. Legal and policy frameworks would need to be developed to ensure that HST reaches those most in need, is conducted in a safe and supported fashion and yields accurate results. Clinical trials may be useful to explore the optimal strategies of introducing HST and to assess its place in a comprehensive HIV prevention package.

We acknowledge that our review has several limitations. First, we used voluntary participation rate as an indicator for the acceptability of the testing strategy. We did not use other indicators such as satisfaction with the testing method, perceived ease of use, difficulties in understanding the testing instructions, satisfaction with pre- and post-test counselling and intention/willingness to use HST [30,43,50-53] because information about these indicators were not available in several of the studies included in the review. We only included studies where participants actively performed HST. Further, only studies published in English were considered. Selection bias is likely as most study populations have been carefully selected and testing experience was generally high. In some studies wanting to perform an HIV test was a precondition for inclusion into the study. Acceptance of HST may be considerably lower among people with less testing experience or for people who did not consider testing for HIV. Overall the quality of the studies included was rather low, included only a small sample of selected participants, and most studies did not compare HST with more traditional testing strategies. Finally, study designs and populations were very heterogeneous and results valid for one country may not be transferrable to another country.

## Conclusions

HST is an acceptable HIV testing strategy for key populations as well as the general population and can be performed accurately by the majority of persons. Linkage to treatment and care services remains a major challenge and different models of HST provision should be explored further. More research is needed to assess feasible and acceptable components of an HST service in order to ensure its safe and reliable use.

## Competing interests

The authors declare that they have no competing interests.

## Authors’ contributions

JK and RC designed the study. JK collected the data, analysed and interpreted the data and drafted and reviewed the manuscript. RC helped to analyse and interpret the data and to draft the manuscript and reviewed all drafts of the manuscript. FSS and CK participated in the interpretation of the data and reviewed drafts of the manuscript. All authors read and approved the final manuscript.

## Pre-publication history

The pre-publication history for this paper can be accessed here:

http://www.biomedcentral.com/1471-2458/13/735/prepub
